# The importance of designing a protector for a preterm and low birth weight infant with ectopia cordis

**DOI:** 10.1002/ccr3.8403

**Published:** 2024-01-03

**Authors:** Takeo Mukai, Atsushi Ito, Yoshihiko Shitara, Kohei Kashima, Mika Kobayashi, Kazuhiro Shiraga, Shinya Takazawa, Naoto Takahashi

**Affiliations:** ^1^ Department of Pediatrics The University of Tokyo Hospital Tokyo Japan; ^2^ Department of Rehabilitation Medicine The University of Tokyo Hospital Tokyo Japan; ^3^ Department of Pediatric Surgery The University of Tokyo Hospital Tokyo Japan

**Keywords:** ectopia cordis, low birth weight infants, preterm, protector

## Abstract

Ectopia cordis is a rare condition with expected low survival rate based on past studies. We encountered a case of a preterm and low birth weight infant with ectopia cordis. When the infant cried, the prolapse of the heart, liver, and intestinal tract worsened. A pressure‐applying protector was used to protect the organs and reduce the prolapse. Upon application, the infant's tachypnea and desaturation worsened. Fluoroscopic examination suggested that the pressure from the prolapsed regions was impeding pulmonary expansion and negatively affecting circulation. It is essential to carefully design a protector that accommodates the infant's growth.

## Graphical abstract

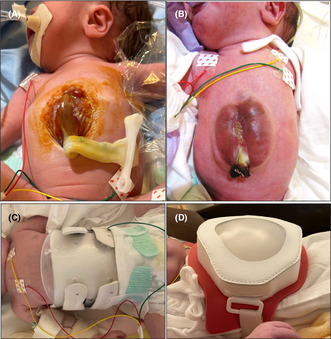

## 1 INTRODUCTION

Ectopia cordis is a rare defect where in a portion or the entire heart is located outside the thoracic cavity, either partially or completely. When the sternum fails to develop, a thoracic wall defect could occur. The annual incidence of ectopia cordis ranges from 5 to 8 per million live births.^1^, ^2^ Ectopia cordis accounts for 0.1% of all heart defects.^3^

Physical protection of the prolapsed organ is extremely important in ectopia cordis; however, there have been no studies on respiratory difficulties resulting from protecting the prolapsed portion of heart in cases of ectopia cordis. We report a case of ectopia cordis in the second infant of twins who was born prematurely and with low birth weight, focusing on the effects of pressure by the protector on respiration.

## 2 CASE REPORT

**FIGURE 1:**
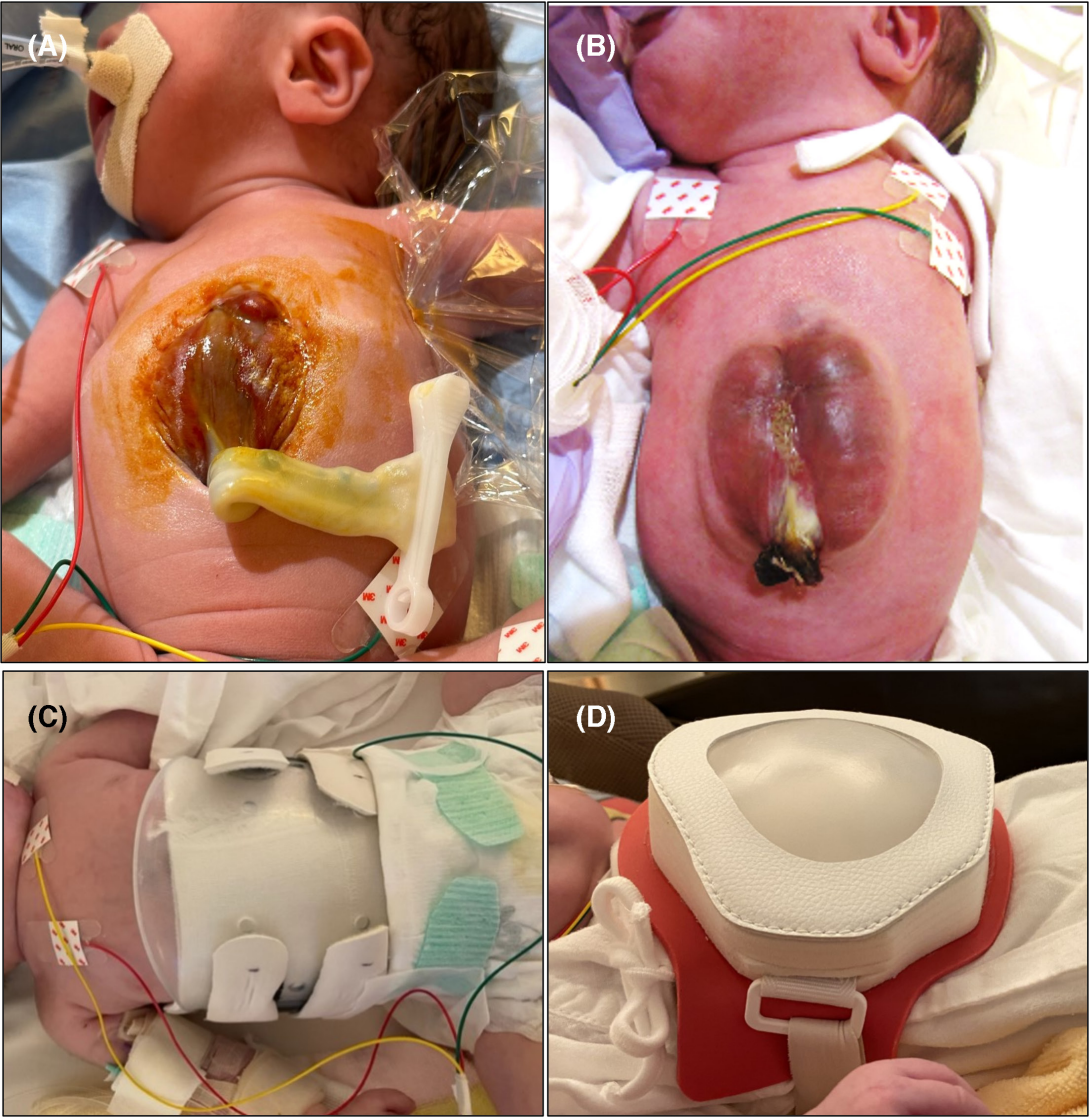
Defect extending from the lower extremity of the chest wall to the abdominal wall, along with an umbilical hernia at birth (A) and at 2 months old (B). A pressure‐applying protector (C) and a protector that did not apply pressure (D) are tried.

The case is that of a preterm and low birth weight infant who was the second twin, suspected of ectopia cordis based on an in‐utero diagnosis. As the first infant experienced a complete rupture of membrane, emergency Cesarean section was performed at a gestational age of 33 weeks and 3 days. The birth weight was 1846 g, and the Apgar score was 8 at 1 min and 8 at 5 min after birth. There was a defect extending from the lower extremity of the chest wall to the abdominal wall, along with an umbilical hernia. As a result, the lower portion of the heart and part of the liver were located outside the body (Figure 1A). Echocardiogram revealed a double outlet right ventricle (DORV), valvular pulmonary stenosis (vPS), and cor triatriatum. During the first two months after birth, the ectopia cordis advanced to epithelization (Figure 1B). When the infant cried, the prolapse of the heart, liver, and intestinal tract worsened (Movie S1). Therefore, to protect the organs and reduce the prolapse, a pressure‐applying protector was used (Figure 1C). Upon application, the infant's tachypnea worsened, and desaturation worsened when the infant cried. Fluoroscopic examination suggested that the pressure from the prolapsed regions was impeding pulmonary expansion and negatively affecting circulation (Movie S2). Subsequently, application of a protector that did not apply pressure resulted in improved respiration and disappearance of desaturation when the infant cried (Figure 1D, Movie S3). On Day 127 after birth, Blalock‐Taussig shunt placement surgery was performed to counter desaturation due to vPS. At 7 months after birth, the infant's body weight was 5 kg, and the infant was discharged from the hospital. In the future, intracardiac repair for DORV, along with radical surgery for the abdominal wall defect and umbilical hernia, is planned. Whether these surgeries are performed simultaneously or sequentially depends on the patient's condition. The protector will be adjusted to suit his growth until the surgery.

## 3 DISCUSSION

Ectopia cordis is a rare condition, occurring in 5–8 births per million.^1^, ^2^ The one‐year survival rate for infants with a body weight of at least 2500 g and gestational age of at least 37 weeks is over 50%.^4^ Survival rates were 4% for infants with birth weight under 1500 g, 6% for those with birth weight 1500–2000 g, 39% for those with birth weight 2000–2500 g, 50% for those with birth weight 2501–3000 g, and 58% for those with birth weight over 3000 g^4^. Survival rates by gestational weeks are 6% for those under 31 weeks, 13% for those at 31–36 weeks, and 59% for those over 37 weeks.^4^ Intracardiac malformation complications have been reported in 8% of infants under 1500 g and in 36% of infants weighing 1500 g or more. The infant in this study had a gestational age of 33 weeks and birth weight of 1846 g. Despite the expected low survival rate based on past studies, the infant experienced uneventful hospital discharge. Additionally, there have been reports of ectopia cordis in twins in the past,^5^ but these were not cases involving preterm infants as in this instance.

Cardiac anomalies associated with ectopia cordis can vary from a single intracardiac defect to the most severe malformation of complete ectopia cordis in combination with more complex intracardiac defects. In a past case series of 31 ectopia cordis cases, 20/31 (64.5%) cases had congenital heart disease; DORV 3/31 (9.7%), ventricular septal defect (VSD) 6/31 (19.3%) tricuspid atresia + vPS, 1/31 (3.2%), pulmonary venous anomalous return 1/31 (3.2%), pulmonary artery enlargement 1/31 (3.2%), truncus arteriosus 1/31 (3.2%), tetralogy of Fallot 2/31 (6.5%), atrioventricular septal defect 2/31 (6.5%), pulmonary atresia + VSD 3/31 (9.7%).^3^ As indicated in this case series, DORV + vPS in this case was not an uncommon complication of congenital heart disease.

Surgical repair is widely acknowledged in the literature as the primary treatment for ectopia cordis. Due to the rarity of this condition, there is no universally accepted best surgical approach.^6^ A multistage repair is the most common approach to surgically treat ectopia cordis as in this case. It is challenging to predict the life prognosis in this case, even if the heart disease is completely cured because of the expected low survival rate associated with premature birth and low birth weight.

Although there have been no studies on respiratory difficulties resulting from protecting the prolapsed portion of heart in cases of ectopia cordis, it is possible that pressure exerted by the prolapsed portion may have an effect on respiration and circulation, as observed in this case. When designing a protector, it is necessary to create a shape that does not interfere with the infant's breathing. Given that newborns, especially preterm or low birth weight infants, experience rapid growth, custom‐made protectors tailored to their growth is important. Therefore, it is essential to carefully design a protector that accommodates the infant's growth.

## AUTHOR CONTRIBUTIONS

**Takeo Mukai:** Conceptualization; funding acquisition; writing – original draft; writing – review and editing. **Atsushi Ito:** Supervision; writing – review and editing. **Yoshihiko Shitara:** Supervision; writing – review and editing. **Kohei Kashima:** Supervision; writing – review and editing. **Mika Kobayashi:** Supervision; writing – review and editing. **Kazuhiro Shiraga:** Supervision; writing – review and editing. **Shinya Takazawa:** Supervision; writing – review and editing. **Naoto Takahashi:** Supervision; writing – review and editing.

## FUNDING INFORMATION

This study was supported by the Japan Society for the Promotion of Science (JSPS KAKENHI Grant Number 23K17432).

## CONFLICT OF INTEREST STATEMENT

The authors declare no conflict of interest.

## CONSENT

Written informed consent was obtained from parents to publish this report in accordance with the journal's patient consent policy.

## Supporting information


Movie S1.

Movie S2.

Movie S3.

Movie Caption.

## Data Availability

Data sharing is not applicable to this article as no datasets were generated or analyzed during the current study.
