# Supplementary material for: The importance of designing a protector for a preterm and low birth weight infant with ectopia cordis
Source: Clin Case Rep. 2024 Jan 3;12(1):e8403. doi: 10.1002/ccr3.8403 (PMC10762480; doi:10.1002/ccr3.8403)
Supplement: Supplementary file 4 — Movie Caption. [file CCR3-12-e8403-s002.docx]

Movie S1 When the infant cried, the prolapse of the heart, liver, and intestinal tract worsened.

Movie S2 Upon a pressure-applying protector application, fluoroscopic examination showed that the pressure from the prolapsed regions was impeding pulmonary expansion.

Movie S3 Application of a protector that did not apply pressure resulted in improved respiration and disappearance of desaturation when the infant cried.
